# Supplementary material for: Neuronal Sequences and dynamic coding of water-sucrose categorization in rat gustatory cortices
Source: iScience. 2024 Oct 30;27(12):111287. doi: 10.1016/j.isci.2024.111287 (PMC11617401; doi:10.1016/j.isci.2024.111287)
Supplement: Document S1. Figures S1–S6 and Tables S1–S3 [file mmc1.pdf]

iScience, Volume *27*

Supplemental information

Neuronal Sequences and dynamic coding of water-sucrose categorization in rat gustatory cortices

Germán Mendoza, Esmeralda Fonseca, Hugo Merchant, and Ranier Gutierrez

Supplementary figures and tables

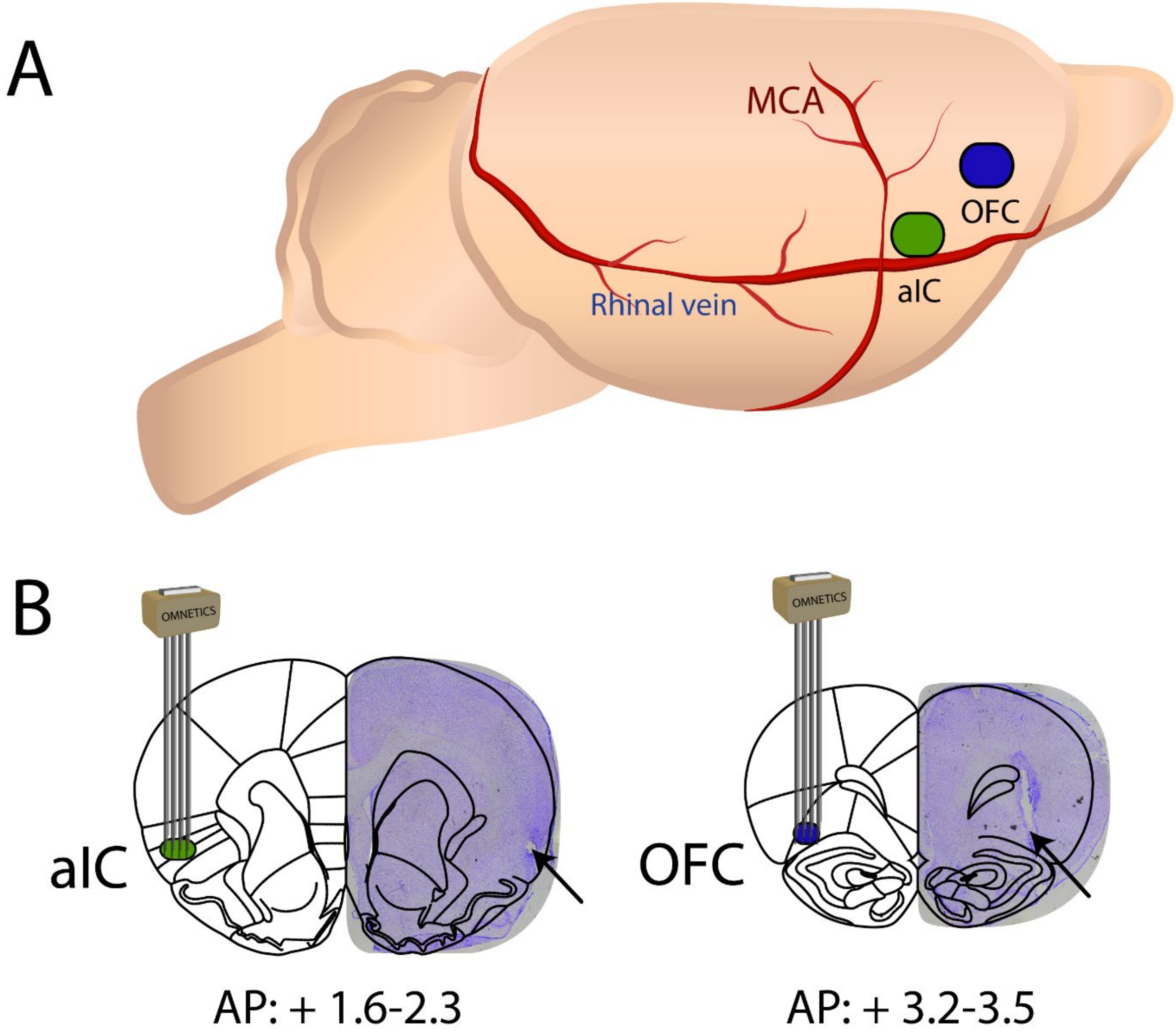

Figure S1. Extracellular recordings were obtained in the anterior Insular cortices (aIC) or the orbitofrontal cortex (OFC). A schematic drawing of the parasagittal view of the brain showing in color the lateral location of each taste cortex recorded, using the medial cerebral artery (MCA) and the caudal rhinal vein as anatomical landmarks. Below is a coronal brain section with a drawing of a 4 × 4, 1 mm2, a homemade 16-electrode array implanted, and a Nissl-stained brain slide showing the electrode tracks overlaid on the Paxinos atlas. Arrows indicate the tip of electrode tracks.

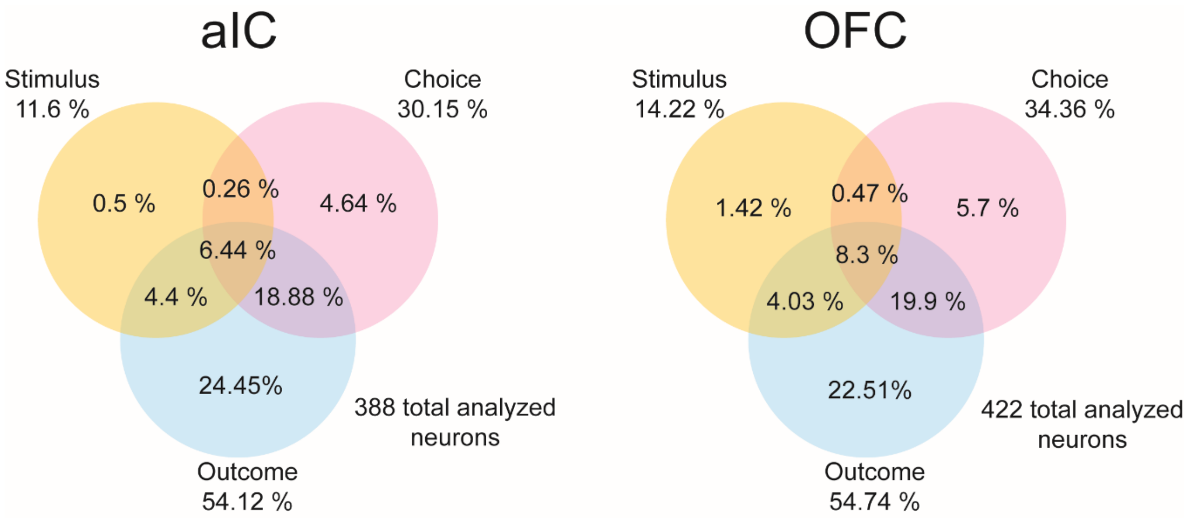

Figure S2. Proportion of encoding neurons from the Signal Detection Theory analysis.

The numbers indicate the percentage of neurons with significant encoding relative to the total number of neurons analyzed for each task variable in aIC (left panel) or OFC (right panel). Cells coding more than one parameter are indicated in the overlapped areas.

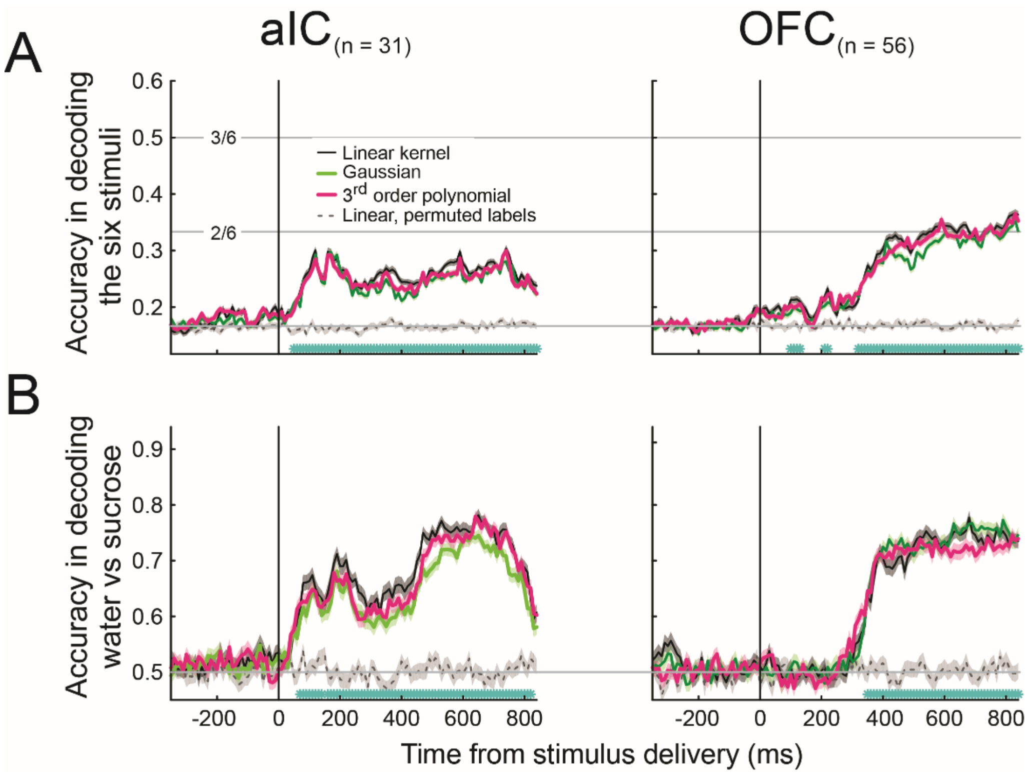

Figure S3. The distinction between water and sucrose, not the identity of the six stimuli, is the strongest taste information encoded in the firing rate of the neural populations in aIC and OFC, related to Figure 3.

(A) Mean ± SEM accuracy from a linear, Gaussian, or third-order polynomial-kernel SVM trained to decode the six different stimuli (0, 0.5, 1.3, 3.2, 7.9, and 20 % sucrose) from the activity of aIC (left panel) and OFC (right panel) neurons. The gray dashed lines show the decoding accuracy from a linear-kernel SVM where the stimulus labels were randomly permuted. Data are aligned with the time of stimulus delivery. Cyan asterisks indicate bins where significant differences between the linear SVM and the linear permuted SVM were found (One tail, t-test, $p <= 0.0001$, Bonferroni corrected). The light gray horizontal lines indicate the decoding performance at 1/6 (chance), 2/6, and 3/6.

(B) Accuracy from SVM trained to decode the delivery of water (0% sucrose) or sucrose (0.5, 1.3, 3.2, 7.9, and 20% sucrose, pooled) stimulus from the activity of the same neurons in panel (**A**). The light-gray horizontal line indicates the decoding performance at the chance (0.5). The ordinate axes in (**A**) and (**B**) show distinct ranges at the same scale. Same conventions as in panel (**A**).

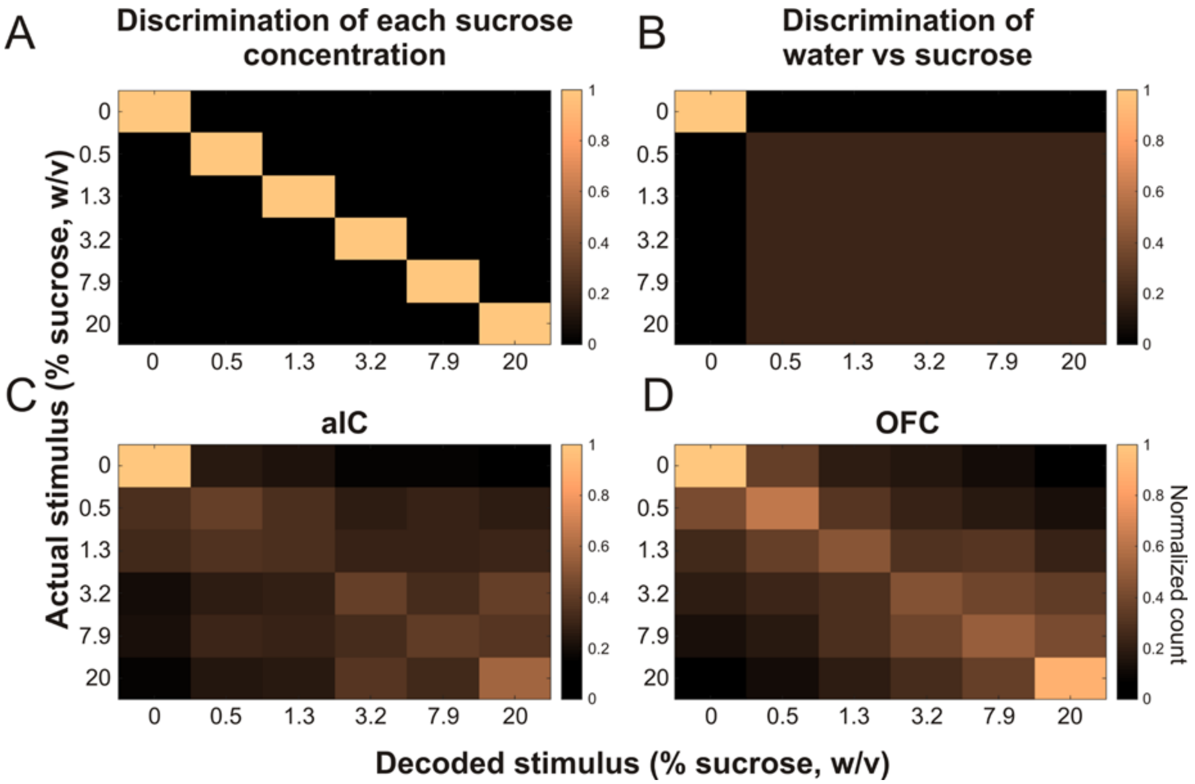

Figure S4. The decoding pattern of aIC and OFC neural activity is more similar to a gross categorization between water and sucrose than to the discrimination of each sucrose concentration, related to Figure 3.

(A) A theoretical confusion matrix showing a perfect discrimination of each sucrose concentration. The matrix shows the theoretical count (see the color code in panel **D**) of the labels assigned by the linear-kernel SVM decoder to each stimulus. The counts are maximal at the corresponding sucrose concentration at the matrix diagonal.

(B) Theoretic pattern of gross categorization between water and sucrose. Pure water is perfectly discriminated, but no clear discrimination between each sucrose concentration is observed.

(C) Responses of the decoder trained with aIC activity. The neural activity did not discriminate each sucrose concentration. The best-discriminated stimuli were pure water and the highest sucrose concentration.

(D) Responses of the decoder trained with OFC activity. The pattern is more similar to the discrimination of each concentration, but specific sucrose concentrations are still ‘confused’ with the lower and the higher ones. The best-discriminated stimuli were pure water and the highest sucrose concentration.

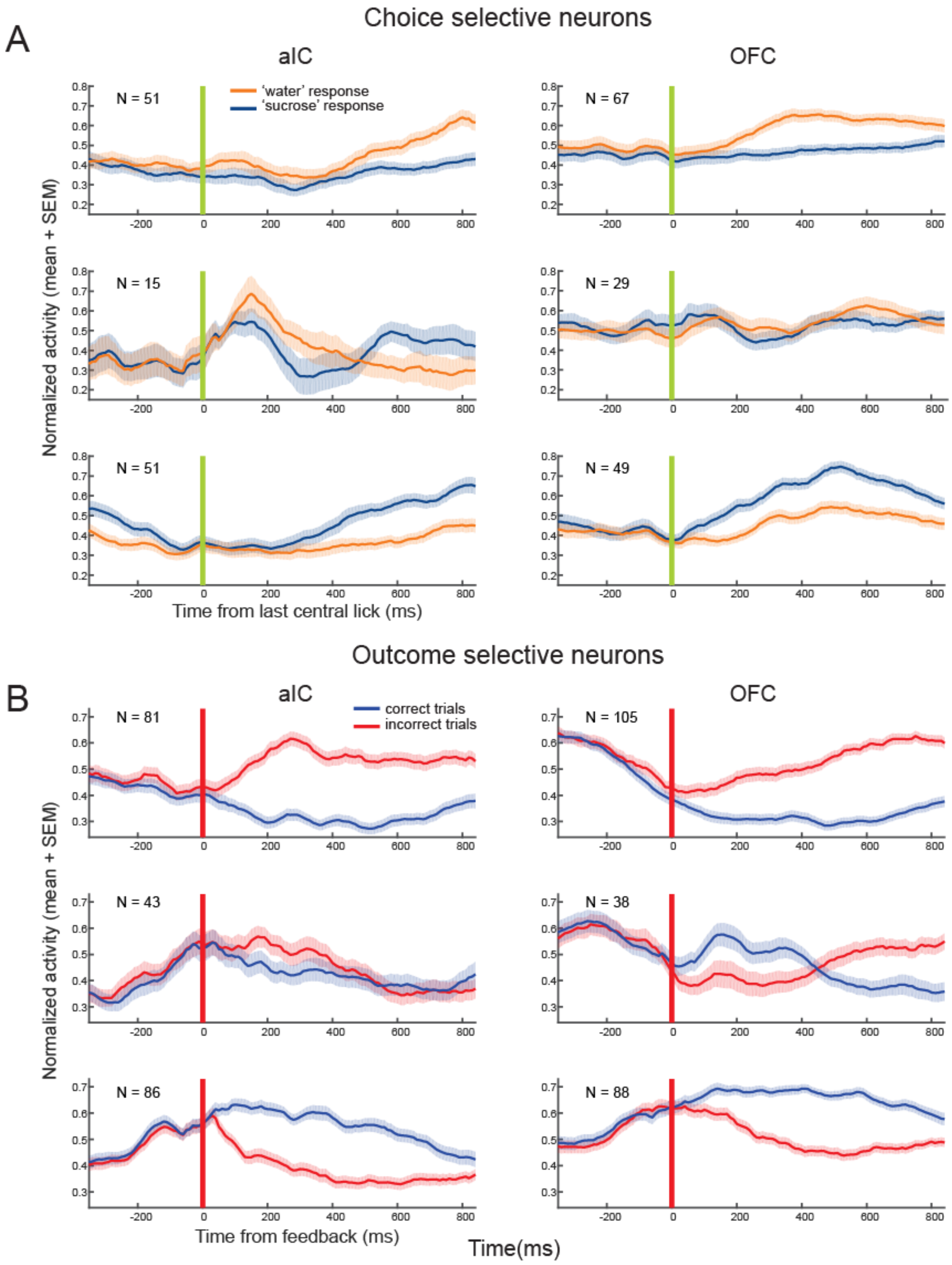

Figure S5. Activity patterns of neurons that encode the rat's decision or the reward outcome, related to Figures 4 and 5.

(A) Normalized population activity (mean $\pm$ SEM) of AIC and OFC neurons selective for the rat's choice. Green vertical lines indicate the time of the last lick in the central port. Neurons were additionally segregated into neurons with higher activity for 'water' responses (upper panel), neurons with higher activity for 'sucrose' responses (lower panel), or neurons with changes in selectivity (intermediate panel). The number of neurons in each sub-population is stated in the graphs.

(B) Analogous segregation was performed on neurons encoding the trial's outcome. Red vertical lines indicate the feedback time.

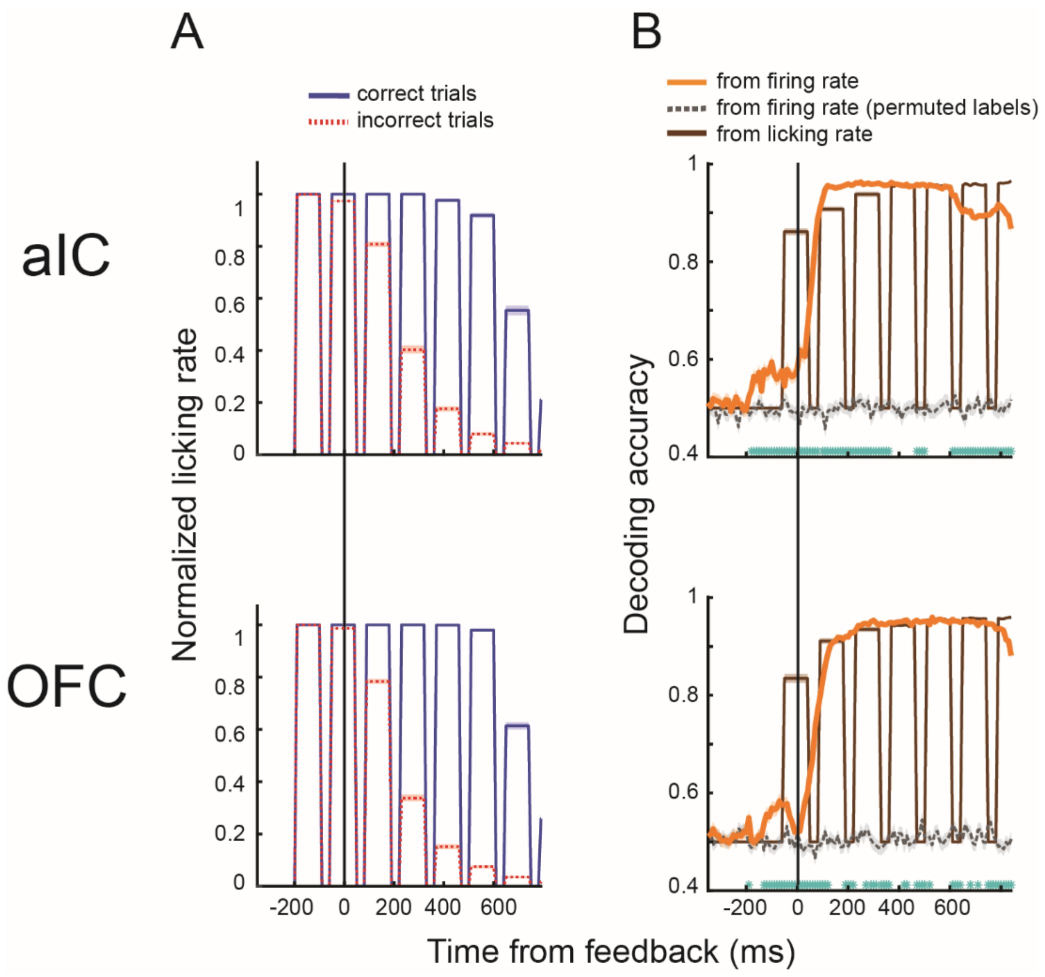

Figure S6. The rat's licking behavior does not entirely explain the encoding properties of the outcome-related neurons.

(**A**) Normalized licking rates (mean + SEM) in rewarded (blue line) and unrewarded trials (red line) from the sessions where aIC (upper panel) or OFC outcome-encoding neurons shown in **Figure 5C** were recorded.

(**B**) Accuracy in decoding rewarded from unrewarded trials (mean + SEM) using either the lick rates of the rats (brown) or the firing rates (orange) of the aIC (upper panel) and OFC neurons (lower panel) shown in the **Figure 5C**. Cyan asterisks mark the bins where the classification accuracy calculated from the firing rate differs from that calculated from lick rates according to a bootstrapping test on the mean differences (see Methods). Note that the licking-related decoding accuracy drops to chance (0.5) periodically, but the decoding accuracy of the neural activity remains high during the post-feedback period.

Supplementary Tables

Supplementary Table 1. Related to Figure 1D. Pair-wise comparisons (Tukey-Kramer test) from results of a two-way ANOVA with sucrose concentration and reward outcome as factors and licking duration in the center spout as the dependent variable. Values are the difference in means. Asterisks indicate significant differences: * < 0.05, ** < 0.01, *** < 0.001.

		Correct trials						Incorrect trials					
		**0 %**	**0.5 %**	**1.3 %**	**3.2 %**	**7.9 %**	**20 %**	**0 %**	**0.5 %**	**1.3 %**	**3.2 %**	**7.9 %**	**20 %**
**Correct trials**	**0 %**		-0.14***	-0.15***	-0.22***	-0.26***	-0.37***	-0.10	-0.06	-0.08	-0.27**	-0.31***	-0.60***
	**0.5 %**			-0.004	-0.07	-0.11*	-0.22***	0.04	0.08	0.05	-0.12**	-0.16***	-0.46***
	**1.3 %**				-0.07	-0.10*	-0.22***	0.04	0.09	0.06	-0.12*	-0.16***	-0.45***
	**3.2 %**					-0.03	-0.14***	0.12**	0.16***	0.13**	-0.04	-0.08	-0.38***
	**7.9 %**						-0.11*	0.15***	0.19***	0.17***	-0.01	-0.05	-0.34***
	**20 %**							0.26***	0.31***	0.28***	0.10	0.05	-0.23***
**Incorrect trials**	**0 %**								0.04	0.01	-0.16***	-0.21***	-0.50***
	**0.5 %**									-0.02	-0.21***	-0.25***	-0.54***
	**1.3 %**										-0.18***	-0.22***	-0.52***
	**3.2 %**											-0.04	-0.33***
	**7.9 %**												-0.29***
	**20 %**												

Supplementary Table 2. Related to Figure 1E. Pair-wise comparisons (Tukey-Kramer test) from results of a two-way ANOVA with sucrose concentration and reward outcome as factors and movement time as the dependent variable. Values are the difference in means. Asterisks indicate significant differences: * < 0.05, ** < 0.01, *** < 0.001.

		Correct trials						Incorrect trials					
		**0 %**	**0.5 %**	**1.3 %**	**3.2 %**	**7.9 %**	**20 %**	**0 %**	**0.5 %**	**1.3 %**	**3.2 %**	**7.9 %**	**20 %**
**Correct trials**	**0 %**		-0.64	-0.13	-0.58	-0.28	-0.13	-1.22	-0.61	-0.72	-3.01**	-2.46*	-6.29***
	**0.5 %**			0.50	0.06	0.35	0.50	-0.58	0.03	-0.08	-2.36	-1.81	-5.65***
	**1.3 %**				-0.44	-0.15	0.00	-1.08	-0.47	-0.58	-2.87**	-2.32	-6.15***
	**3.2 %**					0.29	0.44	-0.64	-0.03	-0.14	-2.43	-1.88	-5.71***
	**7.9 %**						0.15	-0.93	-0.32	-0.43	-2.72*	-2.17	-6.00***
	**20 %**							-1.09	-0.47	-0.58	-2.87**	-2.32	-6.16***
**Incorrect trials**	**0 %**								0.61	0.50	-1.78	-1.23	-5.07***
	**0.5 %**									-0.11	-2.40	-1.84	-5.68***
	**1.3 %**										-2.28	-1.73	-5.57***
	**3.2 %**											0.55	-3.28**
	**7.9 %**												-3.83***
	**20 %**												

Supplementary Table 3. Related to Figure 1F. Pair-wise comparisons (Tukey-Kramer test) from results of a two-way ANOVA with sucrose concentration and reward outcome as factors and lateral licking duration as the dependent variable. Values are the difference in means. Asterisks indicate significant differences: * < 0.05, ** < 0.01, *** < 0.001.

		Correct trials						Incorrect trials					
		**0 %**	**0.5 %**	**1.3 %**	**3.2 %**	**7.9 %**	**20 %**	**0 %**	**0.5 %**	**1.3 %**	**3.2 %**	**7.9 %**	**20 %**
**Correct trials**	**0 %**		0.02	0.02	0.02	0.01	0.003	0.59***	0.49***	0.51***	0.56***	0.57***	0.58***
	**0.5 %**			-0.001	0.004	-0.003	-0.01	0.57***	0.47***	0.49***	0.54***	0.55***	0.56***
	**1.3 %**				0.005	-0.002	-0.01	0.57***	0.47***	0.49***	0.54***	0.55***	0.56***
	**3.2 %**					-0.007	-0.02	0.56***	0.46***	0.49***	0.53***	0.55***	0.56***
	**7.9 %**						-0.01	0.57***	0.47***	0.50***	0.54***	0.55***	0.57***
	**20 %**							0.59***	0.48***	0.51***	0.55***	0.57***	0.58***
**Incorrect trials**	**0 %**								-0.10***	-0.07**	-0.03	-0.01	-0.004
	**0.5 %**									0.02	0.07*	0.08**	0.09***
	**1.3 %**										0.04	0.05	0.07*
	**3.2 %**											0.01	0.02
	**7.9 %**												0.01
	**20 %**												
